# Regulation of viral hepatitis by N6-methyladenosine RNA methylation

**DOI:** 10.1080/22221751.2025.2544726

**Published:** 2025-08-12

**Authors:** Jae-Su Moon, Aleem Siddiqui, Geon-Woo Kim

**Affiliations:** a Center for Rare Disease Therapeutic Technology, Therapeutics & Biotechnology Division, Korea Research Institute of Chemical Technology (KRICT), Daejeon, Korea; b Division of Infectious Diseases and Global Public Health, Department of Medicine, University of California San Diego, La Jolla, USA; c Department of Microbiology & Molecular Biology, Chungnam National University, Daejeon, Korea

**Keywords:** Hepatitis B virus, hepatitis C virus, hepatitis Delta virus, N6-methyladenosine RNA methylation, viral hepatitis

## Abstract

Recent studies have shown that the presence of an RNA modification, N6-methyladenosine (m^6^A), in viral RNAs during infection significantly impacts the outcome of viral replication and pathogenesis. In particular, various functions of m^6^A have been elucidated in hepatitis B virus (HBV), hepatitis C virus (HCV), and hepatitis delta virus (HDV). During viral infection, m^6^A methylation not only directly affects the replication of these viruses but also regulates diverse cellular RNAs to control pathogenesis. This review aims to explore the functions of m^6^A modification in the infectious processes and pathogenesis of HBV, HCV, and HDV. Understanding the role of m^6^A methylation in these viral life cycles is essential for elucidating their pathogenesis and developing novel therapeutic strategies for HBV, HCV, and HDV infections.

## 1. Introduction

Hepatitis B virus (HBV), hepatitis C virus (HCV), and hepatitis Delta virus (HDV) are among the most significant causes of chronic hepatitis worldwide [1–3]. These viruses chronically infect hepatocytes, leading to progressive liver diseases such as fibrosis, cirrhosis, and, ultimately, hepatocellular carcinoma (HCC), the most common type of primary liver cancer. Despite the availability of an effective vaccine against HBV, approximately 350 million people worldwide are infected with the virus, resulting in ∼600,000 deaths reported annually [1]. While antiviral agents for HBV infection effectively suppress viral replication, they do not eliminate the virus completely. Following infection, the HBV genome is converted to covalently closed circular DNA (cccDNA) within the nucleus, which is not completely cleared by current antiviral drugs [4]. Consequently, once the treatment of antiviral medications is discontinued, HBV replication can reinitiate from cccDNA. The inability to completely eradicate HBV infection remains a significant challenge today. In the case of HCV infection, it can be effectively controlled with direct-acting antivirals (DAAs) [5]. However, current DAAs are not effective against all HCV genotypes, and even after successful treatment, individuals cured of HCV may still face the risk of liver disease progression, including the development of HCC [6]. HDV is a defective virus that relies on the HBV life cycle for its replication. It is estimated that at least 25 million people are chronically co-infected with both HBV and HDV [3]. HDV superinfection in individuals already chronically infected with HBV leads to increased liver damage, accelerating the progression of liver disease, leading to cirrhosis, liver failure, and HCC [7]. Currently, no effective antiviral treatment exists for chronic HDV infection.

**Figure 1.**
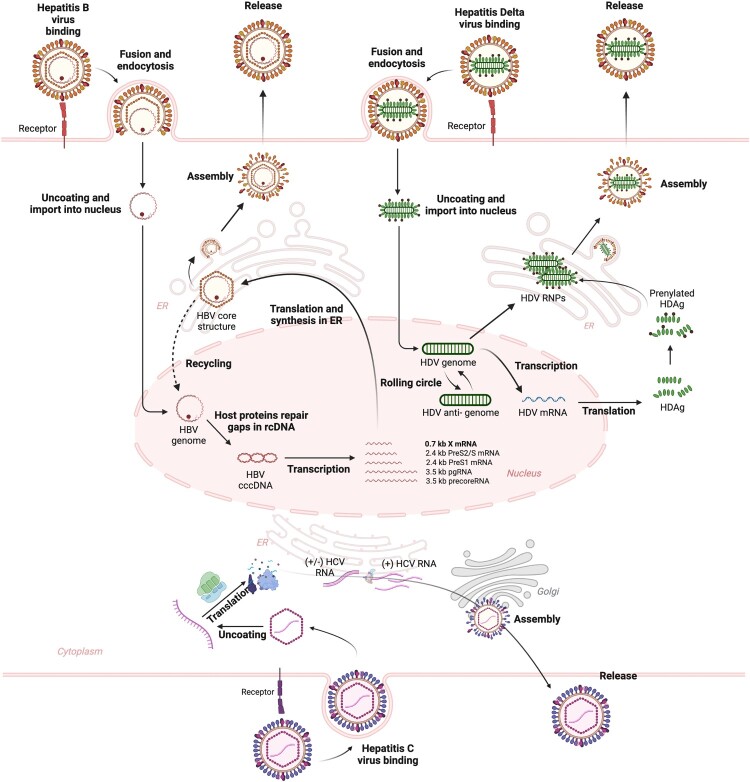
Overview of the life cycles of HBV, HCV, and HDV. HBV life cycle: HBV virions infect human hepatocytes by binding to the sodium taurocholate co-transporting polypeptide (NTCP) receptor, mediated by the epidermal growth factor receptor (EGFR) as a co-receptor. After infection, the HBV nucleocapsid is transported to the nuclear membrane, releasing its relaxed circular DNA (rcDNA) into the nucleus. Within the nucleus, the rcDNA genome is converted into cccDNA by viral polymerase and host factors. The cccDNA is highly resistant to host antiviral responses and can persist in hepatocytes for long periods, contributing to chronic infection. HBV cccDNA encodes the viral proteins: surface (HBs), pre-core or e (HBe), and core (HBc) antigen, polymerase, and X (HBx). The viral polymerase interacts with pgRNA to initiate nucleocapsid assembly, and pgRNA serves as a template to produce rcDNA within nucleocapsid by reverse transcription. The mature HBV capsid either re-enters the nucleus to maintain cccDNA or is secreted from the cell, acquiring an envelope composed of viral envelope proteins embedded in the host cell lipid membrane during the process. HCV life cycle: The infection of HCV into hepatocytes is mediated by several receptors, including CD81, scavenger receptor class B type (SR-BI), and claudin-1. After attachment, HCV virions enter the cell via clathrin-mediated endocytosis. HCV RNA is then directly translated into a single polyprotein precursor, which is cleaved into three structural and seven nonstructural proteins by viral and host proteases. The non-structural proteins, including NS3/4A, NS5A, and NS5B, are involved in viral replication. HCV RNA replication occurs in membrane-associated complexes derived from the ER, known as the membranous web. The viral polymerase, NS5B, possesses RNA-dependent RNA polymerase activity, enabling it to synthesize negative-sense viral RNA using a positive-sense HCV RNA template. Subsequently, a positive-sense HCV RNA is synthesized from a negative-sense RNA by NS5B, and a new viral RNA genome is packaged into the virions. HDV life cycle: HDV, a defective virus, relies on the HBV life cycle for its propagation. HDV uses the envelope proteins of HBV to egress from and re-enter hepatocytes, indicating that HDV enters the hepatocytes through the same NTCP receptor as HBV. After infection, HDV releases its ribonucleoprotein into the cytoplasm, from where it is transported to the nucleus. HDV replicates using RNA-directed RNA synthesis, producing an anti-genome entirely complementary to the HDV genome, but does not translate antigens. This anti-genome is used as a template to produce the HDV genome. This replication occurs via host RNA polymerase II through a rolling-circle mechanism facilitated by self-cleaving RNA sequences (ribozymes). HDV mRNAs are synthesized from the HDV genome and translated at the ER to produce HDAg proteins, which then re-enter the nucleus to enhance genome replication. Small and large HDAg (S-HDAg and L-HDAg) proteins associate with newly synthesized genomic RNA to form new RNPs. These RNPs are transported back to the cytoplasm, where L-HDAg aids in linking to HBV surface antigen (HBsAg) in the ER, leading to the assembly of new viral particles. These viral particles are then released from hepatocytes via the Golgi apparatus to infect adjacent cells.

HBV, a member of the *Hepadnaviridae* family, carries a 3.2 kb partially double-stranded relaxed circular DNA (rcDNA) genome (Figure 1) [1]. HBV infects human hepatocytes by binding to the sodium taurocholate co-transporting polypeptide (NTCP) receptor, with the epidermal growth factor receptor (EGFR) serving as a co-receptor (Figure 1) [8,9]. After infection, the HBV nucleocapsid is transported to the nuclear membrane, releasing its relaxed circular DNA (rcDNA) into the nucleus [1]. Within the nucleus, the rcDNA genome is converted into cccDNA by viral polymerase and host factors. The cccDNA is highly resistant to host antiviral responses and can persist in hepatocytes for long periods, contributing to chronic infection [4]. HBV cccDNA encodes several viral proteins: surface (HBs), pre-core or e (HBe), and core (HBc) antigen, polymerase, and X (HBx) proteins [1]. Although HBV is a DNA virus, its replication involves an RNA intermediate known as pregenomic RNA (pgRNA) [1]. The viral polymerase interacts with 5’ epsilon (ϵ) of pgRNA to form a polymerase-pgRNA complex, followed by recruitment of core protein to initiate pgRNA encapsidation. Within the nucleocapsid, pgRNA serves as a template for rcDNA synthesis via the reverse transcription activity of the viral polymerase.

HCV is a positive-sense single-stranded RNA virus that belongs to the *Flaviviridae* family [2]. HCV genome is about 9.6 kb long and highly structured, containing a single open reading frame (ORF) encoding a polyprotein. HCV entry into hepatocytes is mediated by several receptors, including CD81, scavenger receptor class B type (SR-BI), and claudin-1 (Figure 1) [2]. After attachment, HCV virions enter the cell via clathrin-mediated endocytosis. HCV RNA is then directly translated into a single polyprotein precursor, which is cleaved into three structural and seven nonstructural proteins by viral and host proteases. HCV translation is facilitated by an internal ribosome entry site (IRES) element located within the 5’-untranslated region (UTR), spanning approximately the first 340 nucleotides (nt) [10]. The initiation of IRES-mediated translation involves multiple structural domains and long-range cis-acting elements [10]. The non-structural proteins, including NS3/4A, NS5A, and NS5B, are involved in viral replication, which occurs in membrane-associated complexes derived from the endoplasmic reticulum, known as the membranous web [2]. The viral polymerase, NS5B, possesses RNA-dependent RNA polymerase activity, enabling it to synthesize negative-sense viral RNA using a positive-sense HCV RNA template [11]. Subsequently, a positive-sense HCV RNA is synthesized from the negative-sense RNA by NS5B, and the new viral RNA genome is packaged into the virions.

HDV is a satellite virus that requires co-infection with HBV for its propagation (Figure 1) [12]. HDV virions contain a ribonucleoprotein (RNP) complex, which consists of about 1.7 kilo-nt circular negative-sense single-stranded RNA genome along with the viral proteins small an large delta antigens (S-HDAg and L-HDAg) [12]. HDV uses the envelope proteins of HBV to egress from and re-enter hepatocytes, indicating that HDV enters the hepatocytes through the same NTCP receptor as HBV. However, HDV genome replication is independent of HBV replication [12]. Upon infection, HDV replicates using RNA-directed RNA synthesis, producing an anti-genome that is entirely complementary to the HDV genome. This anti-genome is not translated into any viral proteins, but instead serves as a template to produce the HDV genome. HDV genome replication is mediated by host RNA polymerase II through a rolling-circle mechanism facilitated by self-cleaving RNA sequences (ribozymes) [13]. HDV mRNAs are synthesized from the HDV genome and translated into S-HDAg and L-HDAg [14]. The HDV genome adopts a quasi-double-stranded RNA structure shielded by RNP complexes bound to HDAg [15]. S-HDAg is essential for genome replication and mRNA transcription, whereas L-HDAg is critical for virus assembly but inhibits genome replication.

**Figure 2.**
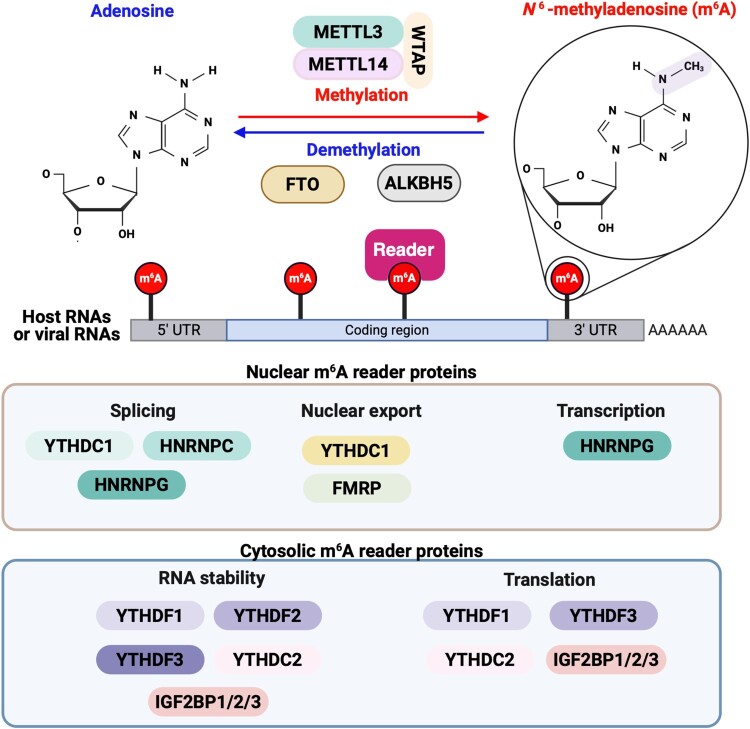
Cellular m^6^A machinery: m^6^A methyltransferases, m^6^A demethylases, and m^6^A reader proteins. m^6^A RNA methylation of the adenosine base at the nitrogen 6 position is the most abundant and well-characterized modification of cellular RNAs. This modification is predominantly enriched in the 5’ and 3'-untranslated regions (UTRs) and adjacent to the stop codon. m^6^A methylation is reversibly catalyzed by m^6^A “writers” (the METTL3, METTL14, and WTAP complex) and removed by “erasers” (FTO or ALKBH5). For the regulation of m^6^A methylation-mediated RNA function, m^6^A “reader” proteins must bind to m^6^A methylated RNA, and then these proteins regulate m^6^A-methylated RNA stability, turnover, and translation.

The functional roles of RNA N6-methyladenosine modification (m^6^A), an emerging topic in RNA biology, are under active investigation. Although discovered in the 1970s, m^6^A methylation could not initially be pinpointed to specific RNA locations due to the technical limitations [16]. Recently, advancements in technology, such as high-throughput sequencing, have allowed for mapping m^6^A distribution across the cellular transcriptome and identifying specific m^6^A sites in RNA [17,18]. m^6^A is the most common internal RNA modification, involving the addition of a methyl group to the adenosine base at nitrogen 6 position. This modification occurs co-transcriptionally within the consensus RRACH/DRACH (D = G, A, or U; R = G or A; H = A, U, or C) sequence (Figure 2) [19]. However, not all DRACH motifs undergo m^6^A methylation. Typically, these modifications are found in the 5’ and 3'-untranslated regions (UTRs) and adjacent to the stop codon and are linked to significant biological processes such as immune response, cell differentiation, circadian clock regulation, sex determination, stress responses, and carcinogenesis [19,20]. The reversible catalysis of m^6^A methylation is mediated by m^6^A methyltransferases (the METTL3, METTL14, and WTAP complex) and demethylases (FTO or ALKBH5) (Figure 2) [20–22]. In addition to the METTL3-METTL14-WTAP complex, other methyltransferase components such as METTL16, zinc finger CCHC-type containing 4 (ZCCHC4), and vir-like m^6^A methyltransferase associated (VIRMA, also known as KIAA1429) have been identified. METTL16 catalyzes m^6^A methylation on U6 snRNA and specific pre-mRNAs, while ZCCHC4 mediates m^6^A modification in 28S rRNA [23,24]. VIRMA functions as a scaffold that recruits the methyltransferase complex to specific sites, particularly within the 3'-UTR and near stop codons, thereby influencing site-specific m^6^A deposition and alternative polyadenylation [23]. For m^6^A methylation-mediated regulation of RNA function, m^6^A “reader” (YTH domain family [YTHDF]) proteins must bind to m^6^A-methylated RNA (Figure 2) [25]. YTHDF proteins then regulate the stability, turnover, and translation of m^6^A methylated RNA. For instance, YTHDF3 binds to m^6^A methylated RNA and recruits YTHDF1 or YTHDF2 to the target RNA [25]. YTHDF1 enhances the translation of m^6^A-methylated mRNA, facilitating protein synthesis [25]. In addition to promoting translation, YTHDF1 has also been reported to stabilize its target mRNAs by preventing their degradation, often in coordination with YTHDF3 [26]. In contrast, YTHDF2 primarily mediates the decay of m^6^A-modified RNAs by recruiting the carbon catabolite repression-negative on TATA-less (CCR4-NOT) deadenylase complex, since YTHDF itself lacks RNase activity [25,27]. YTH domain-containing proteins 1 (YTHDC1) participates in the nuclear export of mRNA in conjunction with nuclear RNA export factor 1 (NXF1) and is also involved in RNA splicing [28]. YTHDC2 possesses an RNA helicase domain and promotes the translation of m^6^A-modified mRNA by binding to the small ribosomal subunit [29]. The helicase function of YTHDC2 is essential for unwinding secondary structures in mRNA, which aids in the process of mRNA translation [30]. In addition, other m^6^A-binding proteins contribute to the regulation of the functions of m^6^A methylated RNA (Figure 2). Insulin-like growth factor 2 mRNA-binding protein (IGF2BP)1, IGF2BP2, and IGF2BP3 recognize m^6^A-modified transcripts through their K homology (KH) domains. These proteins promote mRNA stability and translation, particularly in the context of oncogene expression [24]. Heterogeneous nuclear ribonucleoprotein C (HNRNPC) and G (HNRNPG), although lacking direct m^6^A-binding motifs, function as indirect readers by recognizing m^6^A-induced structural changes and regulating alternative splicing and RNA processing [24]. Fragile X mental retardation protein (FMRP), another m^6^A reader, binds to methylated mRNAs and modulates neuronal mRNA transport and translation [31]. Consequently, the activities of m^6^A-methylated RNA are epigenetically regulated through interactions with various m^6^A reader proteins.

Several recent studies have emphasized the significance of m^6^A modification in the genomes of RNA viruses and transcripts of DNA viruses [32–34]. m^6^A methylation can impact viral life cycles in multiple ways. The occurrence of m^6^A methylation in viral RNAs directly regulates viral replication through interactions with various m^6^A-binding proteins [32–34]. Furthermore, m^6^A modification can indirectly influence viral replication and pathogenesis by modulating the expression of specific genes crucial for the viral life cycle [35,36]. Understanding the intricate relationship between m^6^A RNA methylation and the life cycles of HBV, HCV, and HDV is essential for elucidating chronic infection mechanisms and guiding the development of novel therapeutic strategies. This review aims to consolidate recent findings on m^6^A in HBV, HCV, and HDV, highlighting how this modification influences viral replication and pathogenesis, and identifying open questions for future research.

## 2. Roles of m^6^A in HBV replication

**Figure 3.**
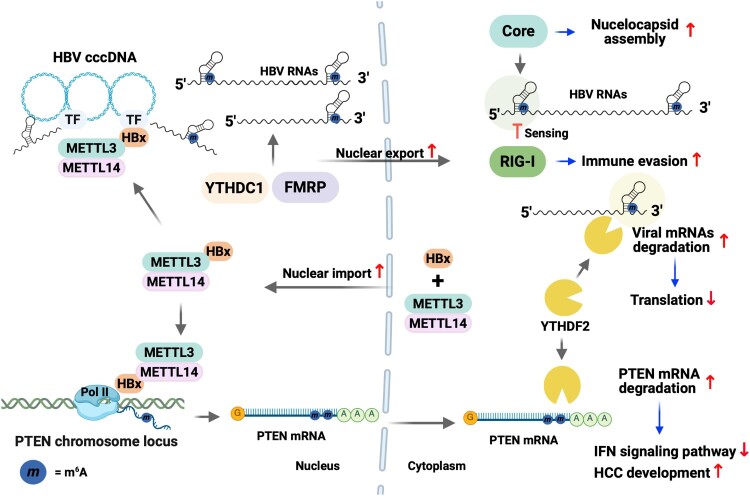
The roles of m^6^A methylation in modulating the HBV life cycle. m^6^A methylation occurs at 1907A, located in the lower stem of the epsilon element. HBV pgRNA includes the epsilon element at both 5’ and 3’ ends due to terminal redundancy, but the other HBV RNAs contain this motif only ate the 3’ end of their viral RNAs. m^6^A methylation at the 5’ epsilon of pgRNA enhances the nucleocapsid assembly by upregulating its interaction with the core. Additionally, this methylation recruits YTHDF2 and 3 inhibits RIG-I sensing to suppress the immune response. Conversely, m^6^A methylation at the 3’ epsilon of HBV RNAs decreases RNA stability by the recruitment of YTHDF2. Moreover, m^6^A modifications at both the 3’ and 5’ epsilon regions facilitate increased nuclear export of the RNA through interactions with YTHDC1 and FMRP. In the context of host genes, m^6^A methylation of PTEN mRNA is increased during HBV infection, and m^6^A methylation of the 3’ UTR of PTEN mRNA decreases its stability via YTHDF2 binding. Reduced PTEN expression attenuates the immune response by inhibiting IRF3 nuclear localization and promotes hepatocarcinogenesis via activation of PI3 K/AKT signalling pathway. HBx enhances nuclear import of METTL3/14 complex and recruits these methyltransferases into HBV cccDNA and the PTEN chromosome locus to add m^6^A RNA methylation.

HBV replication is intricately regulated by m^6^A methylation, which occurs at multiple sites on viral transcripts (Figure 3) [37–42]. Notably, this methylation is predominantly found at adenosine 1907 (A1907), located within the lower stem of the epsilon structure (Table 1) [37]. pgRNA contains the epsilon structure at both the 5’ and 3’ ends due to its terminal redundancy, whereas other viral RNAs carry the epsilon structure only at the 3’ end [1]. Thus, the m^6^A sites are present at both the 5’ and 3’ ends of HBV pgRNA, whereas other HBV transcripts contain the m^6^A site only at the 3’ terminus. The depletion of METLL3 and 14 reduces HBV core-associated DNA levels while enhances the viral RNA stability and translation [37]. The mutation at the m^6^A site of the lower stem of the 3’ epsilon increases viral RNA stability but does not affect core-associated DNA levels. Consistent with these results, the silencing of YTHDF2 enhances HBV RNA stability and protein expression. These observations suggest that YTHDF2 is recruited to the m^6^A methylation site in the lower stem of the 3’ epsilon, reducing viral RNA stability and inhibiting viral protein expression. Conversely, the mutation at the m^6^A site in the lower stem of the 5’ epsilon increases HBV core-associated DNA levels without affecting viral RNA stability and protein expression. Importantly, the occurrence of m^6^A modification at the lower stem of the 5’ epsilon promotes the interaction of pgRNA with core protein and enhances pgRNA encapsidation, leading to the synthesis of rcDNA [40]. These results imply that the m^6^A modification at the 5’ epsilon promotes a more favourable RNA secondary structure that facilitates the interaction with core proteins, thereby enhancing nucleocapsid assembly and the reverse transcription of pgRNA. Thus, m^6^A methylation at the A1907 residue differentially regulates the HBV life cycle depending on its location (Figure 3). In addition to its roles in RNA stability and nucleocapsid assembly, m^6^A modification at position A1907 is also involved in the nuclear export of HBV transcripts (Figure 3) [41]. Silencing of METTL3/14 increases the accumulation of HBV RNAs within the nucleus, suggesting that m^6^A methylation of HBV RNAs is required to facilitate their nuclear export. The nuclear export of m^6^A-methylated HBV RNAs is mediated by m^6^A reader proteins such as YTHDC1 and FMRP, which interact with HBV RNA and promote cytoplasmic localization of HBV transcripts.

**Table 1.** Summary of m^6^A RNA methylations of hepatitis viruses and their functional roles in viral replication.

Virus	m^6^A-methylated site (s)	Functions	References
HBV	5’ epsilon of pgRNA	Increased nucleocapsid assembly & reverse transcription	[37,40,43]
	5’ epsilon of pgRNA	Inhibited RIG-I sensing	[38,43]
	3’ epsilon of all HBV RNAs	Decreased viral RNA stability	[37,43]
	5’ epsilon pgRNA/3’ epsilon of all HBV RNAs	Induced ISG20 binding and degradation of viral RNAs	[39]
	HBx coding region	Decreased HBx RNA stability	[42,43]
HCV	IRES region	Enhanced IRES-mediated translation	[44]
	E1 coding region	Inhibited virion assembly	[45]
	NS5B coding region/3’ UTR	Inhibited RIG-I sensing	[38]
HDV	Not precisely mapped	Decreased virion assembly	[46]

Indeed, additional m^6^A methylation sites of HBV RNA within the region from 1606 to 1809 nt, including five consensus DRACH motifs, have been identified (Table 1) [42]. This region corresponds to the coding region of HBx mRNA and the 3’ UTR of other viral mRNAs. Among the m^6^A methylation sites identified within nt 1606–1809, m^6^A modification at the adenosine of nt 1616 reduces HBx mRNA and protein levels, but does not affect other viral mRNA and protein expressions. These observations suggest that m^6^A methylation within the HBx ORF downregulates HBx protein expression during HBV infection. HBx is known to positively regulate viral gene transcription from cccDNA by interacting with host transcription factors and coactivators [47]. However, reduced HBx protein expression is observed in HBV transfections, transgenic mice, and liver biopsy samples from chronic HBV patients [1,4,48]. Thus, this downregulation of HBx expression by m^6^A modification could have implications for establishing chronic HBV infection.

Notably, HBx plays a vital role in m^6^A methylation deposition on HBV transcripts (Figure 3) [43]. HBx directly interacts with METTL3/14 and subsequently induces the nuclear import of METTL3/14. Within the nucleus, HBx guides METTL3/14 into cccDNA and promotes the addition of m^6^A modification to HBV RNAs during transcription. In the absence of HBx expression, HBV RNAs fail to acquire m^6^A methylation in both HBV-transfected and infected cells. However, introducing exogenous HBx induces m^6^A methylation in viral RNAs, except when HBx lacks a nuclear import signal, suggesting that HBx modulates viral replication through the regulation of m^6^A methylation. Together, these observations demonstrate that m^6^A methylation plays a multifaceted role in HBV replication by modulating viral RNA stability, nucleocapsid assembly, nuclear export of viral RNA, and HBx expression.

## 3. Roles of m^6^A in HCV replication

**Figure 4.**
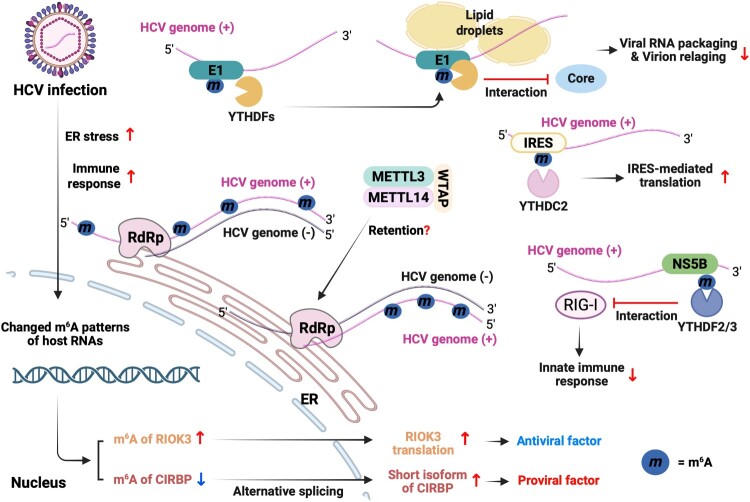
The roles of m^6^A methylation in modulating the HCV life cycle. m^6^A methylation occurs at multiple sites across the HCV genome and plays a significant role in the viral life cycle. Specifically, m^6^A methylation within the IRES region increases IRES-dependent translation by recruiting YTHDC2. In addition, m^6^A methylation in E1 coding region decreases virion assembly by the down-regulation of interaction between the HCV genome and core, while methylation in the NS5B coding region suppresses innate immune response via the abolishing of the viral genome sensing by RIG-I. During HCV infection, alterations in m^6^A methylation also occur in host mRNAs, affecting various cellular processes. These changes in cellular signalling pathways can lead to either an increase or a decrease in HCV replication and contribute to the development of HCC.

m^6^A methylation occurs in approximately 19 regions of HCV RNA, and YTHDF1-3 proteins interact with these m^6^A-methylated regions of the HCV genome [45]. In particular, m^6^A methylation in the region encoding envelope protein 1 (E1) and IRES significantly impacts HCV replication (Figure 4) (Table 1) [44, 45]. Depletion of METTL3/14 in HCV-infected cells increases the production of infectious HCV particles, and a similar result is observed in YTHDF1-3 depleted cells [45]. Conversely, silencing of m^6^A demethylase FTO decreases the production of HCV infectious virion. During HCV infection, YTHDF1-3 proteins are redistributed to lipid droplets, where HCV virion assembly occurs. The interaction of m^6^A-methylated HCV RNA with YTHDF proteins causes the accumulation of the viral genome in lipid droplets, thereby inhibiting nucleocapsid assembly. Mutations at m^6^A methylation sites within the E1 region significantly increase HCV virion production by enhancing the interaction of the core protein with HCV RNA. These results suggest that the interaction of m^6^A modification in the E1 region interferes with core proteins binding to the viral RNA genome by recruiting YTHDF proteins. Thus, reduced m^6^A methylation in the E1 region facilitates the packaging of HCV genome into nascent viral particles.

m^6^A methylation also plays an important role in HCV IRES-mediated translation, which is essential for the viral life cycle (Figure 4) (Table 1) [44]. The HCV IRES is a highly structured RNA element that allows the ribosome to directly bind to the viral RNA and initiate translation without the typical cap structure [49,50]. The HCV IRES region carries several m^6^A methylation sites [45]. Among these, m^6^A modification at nt 331, located about seven nt upstream of the initiator AUG codon, positively affects IRES-mediated translation [44]. This m^6^A methylation is recognized by YTHDC2, the only YTH domain family protein that contains a helicase domain [30]. The interaction between YTHDC2 and m^6^A methylation at nt 331 of HCV genome increases IRES-mediated translation by unwinding its secondary structure [44]. These data imply that YTHDC2 affects the secondary structure of the IRES region, thereby facilitating ribosome recruitment and translation initiation. This unwinding of the IRES secondary structure is critical because the highly structured HCV IRES can impede ribosome binding and translation initiation. Importantly, YTHDC2 interacts with the La antigen, which is essential for HCV IRES-mediated translation [51,52]. The La antigen assists in placing the AUG start codon of the HCV genome at the entry site of the 40S ribosomal subunit [52]. m^6^A methylation at nt 331 of HCV RNA mediates the interaction between YTHDC2 and the La antigen, promoting La antigen-dependent IRES translation [44]. Mutation at the m^6^A residue at nt 331 abolishes the interaction between YTHDC2 and the La antigen, and IRES-mediated translation is not restored even when La antigen is overexpressed. These results demonstrate that the m^6^A methylation at nt 331 recruits YTHDC2, which then assists in HCV IRES translation initiation through interaction with the La antigen. Additionally, m^6^A methylations have been identified in the RNA genomes of other *Flaviviridae* viruses, such as Zika virus (ZIKV), dengue virus (DENV), yellow fever virus (YFV), and West Nile virus (WNV) [45]. The presence of m^6^A in conserved regions across these viral genomes suggests that m^6^A may serve as a general regulatory mechanism within this virus family.

Generally, m^6^A methylation of cellular mRNA is co-transcriptionally mediated by METTL3/14 in the nucleus, where the METTL3/14 complex associates with chromatin and RNA polymerase II to methylate RNA (Figure 3) [53]. However, HCV replication occurs in the cytoplasm, independent of RNA polymerase II (Figure 4) [2]. Notably, METTL3 and 14 are localized in both the cytoplasm and nucleus [20]. Silencing of METTL3/14 dramatically reduces m^6^A methylation levels of HCV RNA, demonstrating m^6^A methylation of HCV genome is mediated by the cytoplasmic METTL3/14 complex [45]. Intriguingly, the function of WTAP has been highlighted in this context. WTAP, a cofactor of the cellular m^6^A methyltransferase complex, interacts with METTL3/14 complex as an accessory protein to add m^6^A methylation to target RNAs [24]. HCV infection up-regulates the cytoplasmic localization of WTAP, while not altering the subcellular distribution of METTL3/14 [54]. Cytoplasmic WTAP is essential for the m^6^A methylation of the HCV RNA genome through its interaction with METTL3/14 complex. Therefore, m^6^A methylation of the HCV RNA genome can be facilitated by cytoplasmic m^6^A methyltransferases, implying that METTL3/14 can post-transcriptionally deposit m^6^A modification on viral RNAs.

## 4. Roles of m^6^A in HDV replication

**Figure 5.**
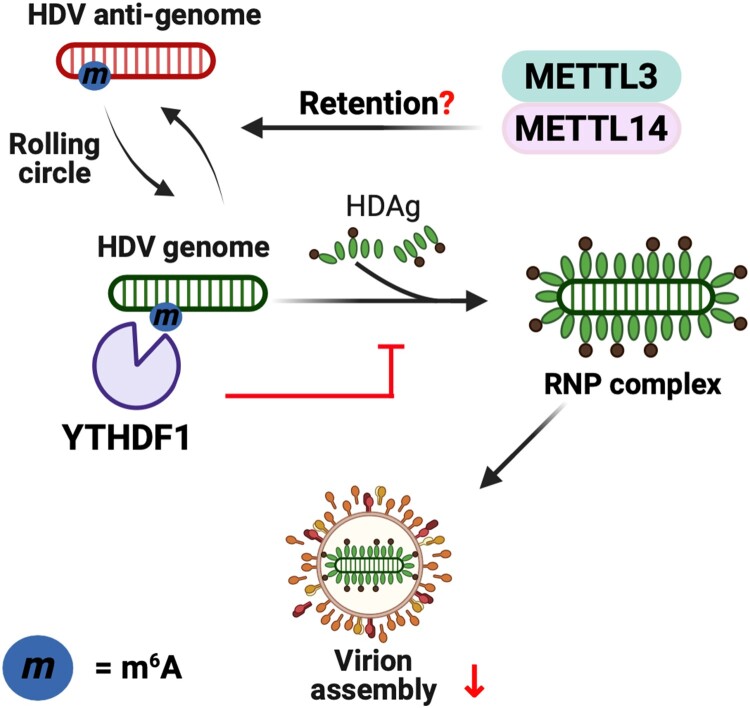
The roles of m^6^A methylation in the regulation of the HDV life cycle. m^6^A methylation in the HDV genome and its impact on virion assembly. m^6^A methylation of the HDV genome recruits YTHDF1. YTHDF1 binding to m^6^A methylated HDV RNA interferes with HDAg binding, thereby reducing RNP formation and virion assembly. Specific m^6^A sites in HDV are not yet identified; the illustration depicts the general effect of m^6^A on HDV RNP formation.

m^6^A methylation occurs in both intracellular HDV genomes and anti-genomes during co-infection with HBV and HDV (Figure 5) (Table 1) [46]. Intriguingly, the extracellular HDV genome retains lower levels of m^6^A methylation than the intracellular HDV genome. m^6^A-methylated HDV genome and anti-genome interact with all YTHDF proteins. Among the YTHDF 1–3 proteins, YTHDF1 shows the strongest interaction with the HDV genome. The absence of METTL3/14 causes a significant decrease in the intracellular HDV genome and HDAg levels, while increasing extracellular HDV genome levels. Consistently, YTHDF1 depletion produces similar effects, including increased extracellular HDV genome levels. m^6^A-dependent binding of YTHDF1 to the HDV genome promotes the accumulation of the HDV genome within cells. Because the binding of HDAg with the HDV genome is essential for virion assembly, the recognition of the genome by YTHDF1 interferes with HDAg interaction, thereby preventing virion assembly (Figure 5) [12,55]. However, specific m^6^A sites in the HDV genome, antigenome, and mRNA have not yet been identified. Indeed, while m^6^A modification occurs in the HDV anti-genome, the function of m^6^A modification in the HDV anti-genome has yet to be explored. Thus, mapping and functionally characterizing m^6^A sites in both the HDV genome and antigenome is essential for understanding the interplay between m^6^A and HDV replication.

## 5. Roles of m^6^A in immune response

Beyond direct effects on viral replication, m^6^A modifications also influence the interplay between these viruses and the host immune system [38,56]. The innate immune response is the first line of defense against viral infections, providing an immediate response to pathogen invasion [57]. This response is primarily mediated by pattern recognition receptors (PRRs), such as toll-like receptors (TLRs) and retinoic acid-inducible gene 1 (RIG-I)-like receptors (RLRs), which recognize the pathogen-associated molecular patterns (PAMP) such as viral double-stranded RNA and 5’ triphosphate RNA [58]. RIG-I binding to PAMP triggers its polyubiquitination, which subsequently facilitates interaction with mitochondrial antiviral signalling protein (MAVS), acting as an adaptor [59]. The interaction between RIG-I and MAVS recruits downstream signalling molecules, including inhibitors of NF-κB kinase (IKK) family members and TNF receptor-associated factor (TRAF)3/6, leading to activation of IRF-3/7 and NF-κb for production of IFN and proinflammatory cytokines. These cytokines negatively control viral replication and spreading while positively affecting the activation of the adaptive immune response [57]. However, various viruses can modulate the innate immune response by multiple mechanisms for establishing persistent infections. In this context, m^6^A RNA methylation has emerged as a crucial regulator of the innate immune response to viral infections [36, 38, 39, 56]. During diverse viral infections, m^6^A modification influences the strength and duration of the innate immune response.

### 5.1. m^6^A-mediated evasion of immune sensing

HBV is considered a stealth virus that establishes persistent infection without activating innate immune response [60]. Although the 5’ epsilon element of HBV pgRNA is recognized by RIG-I, HBV employs multiple strategies to suppress RIG-I-mediated innate immune responses through various mechanisms [61]. One of the mechanisms has been attributed to HBx, which promotes the addition of linear ubiquitin chains to MAVS via the linear ubiquitin assembly complex (LUBAC), thereby disrupting downstream signalling [62]. RIG-I also recognizes the HCV RNA genome to trigger immune response, but viral NS3/4 protease cleaves MAVS to block the production of IFN [63]. Recent studies have highlighted the role of m^6^A RNA methylation in immune evasion during HBV and HCV infections [38]. m^6^A modifications on both HBV transcripts and the HCV genome inhibit their recognition by RIG-I, thereby reducing the IFN signalling pathways. For HBV, m^6^A methylation within the lower stem of the 5’ epsilon element prevents the sensing of pgRNA by RIG-I (Figure 3). In the case of HCV, m^6^A RNA modification occurs within the 3'-end poly (U/UC) region, which is a high-affinity RIG-I ligand (Figure 4). This m^6^A modification reduces RIG-I recognition of HCV RNA, as observed in the case of HBV pgRNA. However, mutations at m^6^A sites within the RIG-I ligand regions of HBV and HCV enhance RIG-I sensing and consequently activate downstream signalling pathways. In this respect, the interaction of the m^6^A reader proteins, such as YTHDF2 and 3, negatively affects RIG-I recognition of m^6^A-methylated viral RNA. Specifically, the binding of YTHDF2/3 to m^6^A-methylated regions of viral RNAs within the RIG-I ligand region suppresses IFN signalling through the down-regulation of RIG-I recognition, which implies that YTHDF proteins and RIG-I competitively bind to viral RNA to regulate the immune response. In addition, m^6^A modification of other viral RNAs, including SARS-CoV-2, inhibits the RIG-I signalling by recruiting YTHDF proteins [64]. Thus, m^6^A-mediated suppression of RIG-I signalling may represent a conserved immune evasion strategy employed by various viruses to promote persistent infection. These findings suggest that viruses may exploit m^6^A methylation to evade the distinction between self and non-self RNA by the immune system. In addition to m^6^A modification, viral RNAs may harbour other chemical modifications, such as 5-methylcytosine, uridine to pseudouridine (U to ψ), and adenosine to inosine (A to I) editing [65]. If these modifications occur within a PRR-recognized region of viral RNA, viruses may exploit them to mimic host RNA and evade innate immune detection.

Additionally, in HBV infection, m^6^A modification influences viral RNA degradation mediated by IFN signalling pathway [39]. In HBV-infected and transfected cells, IFN-α treatment induces the expression of ISG20 exonuclease, which suppresses HBV replication by degrading viral RNAs [66]. ISG20 binds to m^6^A-modified HBV transcripts and promotes their degradation, whereas non-methylated viral RNA resists ISG20 exonuclease activity [39]. YTHDF2 plays a central role in this regulatory mechanism. YTHDF2 interacts with ISG20 and then guides ISG20 onto m^6^A-methylated viral RNA. Together, these results suggest that m^6^A methylation leads to selective degradation of viral RNA by ISG20. In addition to HBV, replication of several viruses, including HCV, WNV, DENV, and Human Immunodeficiency Virus (HIV), is reduced by IFN-α-induced ISG20 exonuclease [67]. Indeed, the RNA genomes of these viruses are m^6^A methylated [34]. These findings suggest that m^6^A methylation can play a similar role in ISG20 mediated degradations of RNA genomes of these viruses as observed in HBV.

### 5.2. Viral manipulation of host immunity via m^6^A-mediated gene expression

Viral infection can affect the expression of host antiviral or proviral genes to evade the host immune response through the modulation of host m^6^A patterns [35,36]. Specifically, HCV infection increases the m^6^A methylation level of cellular RIO kinase 3 (RIOK3) mRNA (Figure 4, Table 2) [36]. The major role of RIOK3 is not only as an SUFU-dependent positive regulator of the Hedgehog signalling pathway but also as an activator of antiviral signalling [69]. Innate immune sensing of the HCV genome as PAMP induces m^6^A modification of RIOK3 mRNA during HCV infection [36]. HCV-induced m^6^A methylation of RIOK3 mRNA enhances its protein expression by promoting interaction with YTHDF1, which in turn suppresses HCV replication. Thus, during HCV infection, RIOK3 protein expression is regulated via m^6^A modification and contributes to the suppression of viral replication by enhancing innate immune signalling. In this study, infections with other Flaviviruses, including DENV, ZIKV, and WNV, have also induced host m^6^A changes similar to those observed in HCV infection, suggesting that these viruses can share the distinct cellular pathways to maintain the persistence of infection by regulating m^6^A patterns of cellular RNA. While increased RIOK3 expression suppresses HCV replication, it enhances the replication of DENV and ZIKV. These results imply that RIOK3 can both enhance and suppress the host immune response by modulating components of the IFN signalling pathway, and that its effects vary in a virus-specific manner. Therefore, elucidating the mechanisms by which RIOK3 regulates the activation or suppression of innate immune responses during viral infection is crucial to understanding host – virus interactions.

**Table 2.** Summary of m⁶A-regulated genes implicated in antiviral immunity and hepatocarcinogenesis in hepatitis virus infections.

Virus	Host gene	m^6^A level	m^6^A-mediated effect	Function	References
HBV	PTEN	Increase	Decreased PTEN mRNA stability	Inhibited innate immune response, Induced HCC	[35]
	circRNA-ARL3	Increase	Increased circRNA-ARL3 stability	Inhibited tumour suppressor function of miR-1305	[68]
HCV	RIOK3	Increase	Increased RIOK3 translation	Enhanced immune response	[36]
	CIRBP	Decrease	Induced short isoform of CIRBP	Induced HCC	[36]

## 6. Role of m^6^A in hepatitis viruses associated with HCC

Viral infections, such as HBV and HCV, generally affect the expression of host genes related to pro- or anti-viral factors, thereby optimizing long-term propagation and contributing to viral pathogenesis [70–73]. In this context, m^6^A RNA methylation acts as a key regulator of virus-induced in host gene expression [35,36].

### 6.1. m^6^A regulation in HBV-mediated HCC

HBV infection regulates the level of m^6^A modification of cellular RNA to evade the immune response, thereby establishing persistent infection and influencing pathogenesis (Table 2) [35]. In this regulatory context, HBx protein is a key factor in the modulation of the m^6^A pattern of the host RNAs (Figure 3) [43]. In addition to regulating the m^6^A methylation of HBV RNAs, HBx also promotes the occurrence of m^6^A modification of host transcripts. For instance, HBx increases m^6^A methylation levels of the phosphatase and tensin homolog (PTEN) transcripts (Figure 3) [35]. PTEN is known as the regulator of the phosphoinositide 3-kinase (PI3 K)/AKT signalling, which reduces cell survival and promotes apoptosis [74]. PI3 K, activated by tyrosine kinase or G-protein-coupled receptors, phosphorylates PIP2 to produce PIP3, which in turn activates the AKT signalling pathway. PTEN, function as a lipid phosphatase, dephosphorylates PIP3 to regenerate PIP2 and thus blocks the PI3 K/AKT signalling cascade, supporting that PTEN plays a tumour suppressor. HBx interacts with METTL3/14 complex in the cytoplasm and promotes their nuclear import [43]. In the nucleus, HBx protein guides METTL3/14 onto the PTEN chromosomal locus to co-trasncriptionally add m^6^A methylation in PTEN mRNA. HBx-induced m^6^A modification of PTEN mRNA increases the interaction with YTHDF2/3 proteins, leading to RNA destabilization. Additionally, PTEN plays an important role in innate immune response [75]. During viral infection, PTEN dephosphorylates the Serine 96 residue of IRF-3 and promotes IRF-3 nuclear translocation to activate the transcription of the genes related to IFN signalling pathways [35]. Consequently, reduced PTEN expression by HBx suppresses the stimulation of IFN synthesis during HBV infection. HBx also affects HCC development via the regulation of microRNA (miR)−1305 function, which suppresses the ubiquitin-conjugating enzyme E2 T (UBE2 T)-mediated AKT-signalling pathway [68]. To regulate miR-1305 function, HBx induces m^6^A modification of circular RNA (circRNA)-ARL3, which recruits YTHDC1 to promote its biogenesis (Table 2). Increased expression of circRNA-ARL3 by HBx induces sequestration of miR-1305, thereby inhibiting the tumour suppressor function of miR-1305 and accelerating HCC development. Therefore, these results suggest that HBx can be involved in HBV-mediated hepatocarcinoma genesis and the evasion of host immune response through the modulation of m^6^A modification of cellular RNAs.

### 6.2. m^6^A regulation in HCV-mediated HCC

Similarly, HCV infection also contributes the development of HCC through the regulation of cellular m^6^A patterns [36]. In particular, induced endoplasmic reticulum (ER) stress by HCV infection decreases the m^6^A levels of cold-inducible RNA-binding protein (CIRBP) mRNA (Figure 4, Table 2). CIRBP encodes the stress-induced RNA binding protein, that regulates reactive oxygens species (ROS) accumulation and contributes the development of HCC (Figure 4). Reduced m^6^A methylation of CIRBP promotes alternative splicing, generating a shorter isoform. Increased the shorter isoform of CIRBP positively affects HCV replication. Expression of CIRBP is upregulated in murine models of hepatocarcinogenesis as well as in liver tissues from HCC patients, where it promotes tumour development by expanding cancer stem/progenitor cell populations through a mechanism involving increased reactive oxygen species and activation of STAT3 signalling. [76]. These results suggest that HCV infection can cause hepatocarcinogenesis via m^6^A-mediated regulation of CIRBP. However, the distinct contributions of the small and long CIRBP isoforms to HCC pathogenesis remain to be clarified. Therefore,further studies are required to elucidate how the small and long isoforms of CIRBP contribute to HCV-related hepatocellular carcinogenesis.

## 7. Conclusion and future prospects

m⁶A RNA methylation has emerged as a pivotal regulatory mechanism in the life cycles of HBV, HCV, and HDV [35–46]. As a dynamic post-transcriptional modification, m⁶A not only governs viral replication and RNA metabolism but also contributes to immune evasion and the pathogenesis of liver disease. Accumulating evidence highlights its virus-specific regulatory roles, suggesting that m⁶A may represent a unifying yet finely tailored mechanism that hepatitis viruses exploit to persist in the host. Understanding these processes offers opportunities to identify novel therapeutic targets and to improve current treatment strategies. In HBV, m⁶A modification is differentially regulating viral life cycle depending on its location [37–40,42]. m⁶A methylation at the 5′ epsilon element facilitates nucleocapsid assembly by promoting pgRNA – core protein interactions and enhancing reverse transcription [40]. Conversely, m⁶A methylation at the 3′ epsilon element reduces RNA stability and viral protein expression [37,40]. These site-specific effects show that m⁶A acts in a highly specific manner to control HBV replication. This suggests that targeting specific m⁶A methylation sites could reduce cccDNA amplification and help overcome viral persistence, which are key barriers to a functional HBV cure. For instance, inhibiting m⁶A methylation at the 5′ epsilon element may interfere with nucleocapsid formation and reverse transcription, thereby reducing HBV replication, while enhancing m⁶A methylation at the 3′ epsilon element could destabilize viral transcripts and suppress viral protein expression. In addition to HBV, HCV also makes use of m⁶A modifications depending on the specific region of the viral RNA, with each site playing a distinct functional role during infection [38,44,45]. The interaction between m⁶A-modified IRES elements and the YTHDC2 reader protein promotes unwinding of RNA secondary structures, thereby enhancing translation initiation [44]. In contrast, methylation in the E1 region attenuates virion assembly through YTHDF1–3-mediated recognition, illustrating the dual role of m⁶A in coordinating efficient replication while limiting excess viral replication [45]. Therapeutically disrupting these post-transcriptional checkpoints could attenuate viral protein synthesis. In HCV infection, for example, this approach may reduce the viral burden by interfering with m^6^A-mediated steps in the viral life cycle. In the case of HDV, viral genomes are also methylated by m⁶A modification, which affects the interaction with hepatitis delta antigen and thereby determines virion assembly [46]. Although specific m^6^A sites of HDV genomes are not yet identified, these modifications hold therapeutic relevance, especially given the strong association of HDV superinfection with accelerated liver fibrosis and HCC.

These m^6^A functions have specific effects on hepatitis viral replication and also influence host RNA biology during viral infection [35,36,43,68]. For instance, the HBV-encoded HBx protein recruits the METTL3/METTL14 complex to chromatin regions of tumour suppressors such as PTEN, facilitating their destabilization via YTHDF2 binding and promoting oncogenic PI3K – AKT signalling [35,43]. In contrast, HCV infection induces m⁶A methylation of RIOK3 mRNA, stabilizing it via YTHDF1 and enhancing antiviral responses [36]. These divergent interactions emphasize the need for comparative studies to elucidate the shared and virus-specific strategies hepatitis viruses use to manipulate the host epitranscriptome. In addition to their impact on viral replication, m⁶A modifications also affect innate immunity during viral infections [38,39,77]. m^6^A methylation of viral RNA at RIG-I recognition sites hinders pattern recognition and interferon signalling, promoting immune evasion [38]. On the other hand, stabilization of key immune effectors such as ISG20 and RIOK3 through m⁶A promotes antiviral activity [36]. These opposing roles underscore the context-dependent nature of m⁶A during infection and point to a fine-tuned balance that viruses exploit to regulate host immune responses. Unravelling the temporal and spatial dynamics of these processes will be key to understanding how viruses establish chronicity.

Despite advances in understanding canonical m⁶A readers such as YTHDF1–3 and YTHDC1/2, the functions of noncanonical readers – such as IGF2BP1–3, HNRNPC, and HNRNPG remain largely unexplored in the context of viral hepatitis. These proteins have been implicated in regulating RNA splicing, stability, and nuclear export in mammalian systems and may play important roles in the cellular response to infection [24]. Comprehensive analyses of RNA – protein interactions and crosslinking-based m⁶A-RNA interactomes will be necessary to reveal how these factors contribute to viral replication, immune evasion, or pathogenesis. Moreover, technological advances in site-specific methylation mapping are essential to move the field forward. While MeRIP-seq has provided foundational insights into m⁶A methylation landscapes, it lacks single-nucleotide resolution. The emergence of more refined platforms, such as m⁶A-SAC-seq, miCLIP2, and nanopore-based direct RNA sequencing, promises greater accuracy and depth, enabling precise mapping of methylation events even in low-abundance viral RNAs [78,79].

From a therapeutic standpoint, targeting the m⁶A methylation pathway offers intriguing potential. METTL3 inhibitors have demonstrated immunomodulatory and antitumour activities in preclinical cancer models, including glioblastoma and acute myeloid leukemia [80]. These compounds promote the accumulation of double-stranded RNA and activate type I interferon signalling, thereby reshaping the tumour-immune microenvironment. The first-in-human clinical trial of a METTL3 inhibitor (STC-15) has revealed immune activation in treated patients, indicating the feasibility of targeting m⁶A pathways in clinical settings [80]. Translating these findings to chronic viral hepatitis could open new avenues for antiviral therapies, especially by enhancing immunogenicity of viral RNAs. Nevertheless, broad targeting of m⁶A enzymes may lead to undesirable consequences for host mRNA metabolism. Therefore, precise and hepatocyte-specific delivery strategies, as well as selective inhibition of virus-specific methylation events, are required to ensure therapeutic efficacy with minimal toxicity. The use of advanced in vitro models, including patient-derived liver organoids and humanized mouse systems, will be instrumental in evaluating the safety and antiviral potential of such agents.

In conclusion, m⁶A methylation represents a critical regulatory layer in hepatitis virus biology, modulating viral replication, host immune responses, and the progression to liver disease. Continued efforts to dissect its complex regulatory roles, in concert with technological innovation and translational application, will be essential to unlock the full therapeutic potential of the epitranscriptome for managing HBV, HCV, and HDV infections.
